# Supplementary material for: Exposure to Inorganic Arsenic in Rice in Brazil: A Human Health Risk Assessment
Source: Int J Environ Res Public Health. 2022 Dec 8;19(24):16460. doi: 10.3390/ijerph192416460 (PMC9778750; doi:10.3390/ijerph192416460)
Supplement: Supplementary file 1 [file ijerph-19-16460-s001.zip › Supplementary Material File S4 - Suplemmentary tables.pdf]

Supplementary Material—Supplementary Tables

Title

“Exposure to inorganic arsenic in rice in Brazil: A human health risk assessment”.

Table S1. Parameters adopted for assessing the risk of exposure to inorganic arsenic in rice.

Age (years)	Body Weight (kg) [36]	Rates of Ingestion Rice (g/day) [13,29,31–33,90]	Exposure Duration (years)
<1	7.82	9.0	0.75/0.50 *
1	11.19	54.9	1.00
2	13.70	54.0	1.00
3	15.71	60.6	1.00
4	17.80	66.7	1.00
5	19.77	58.2	1.00
6	22.17	60.2	1.00
7	24.98	60.2	1.00
8	27.70	60.2	1.00
9	31.66	60.2	1.00
10	33.82	56.2	1.00
11	38.11	56.2	1.00
12	43.06	56.2	1.00
13	47.66	56.2	1.00
14	51.21	56.2	1.00
15	54.87	56.2	1.00
16	56.82	56.2	1.00
17	58.79	56.2	1.00
18	60.38	56.2	1.00
19	61.18	58.4	1.00
20 to <25	63.77	58.4	5.00
25 to <30	66.70	58.4	5.00
30 to <35	67.95	58.4	5.00
35 to <45	68.94	58.4	10.00
45 to <55	69.54	58.4	10.00
55 to <65	68.93	53.7	10.00
65 to <70	66.52	49.1	10.00

* According to the São Paulo Municipal Department of Education guidelines for food consumption at daycare centers, infants should not be fed rice until 4 months of age (São Paulo, 2011a); and the WHO recommends that infants start to drink water at 6 months of age [90].

Table S2. Descriptive statistics for inorganic arsenic (iAs) concentrations in samples of polished (white) and husked (brown) rice.

Statistic	White rice (n = 64) (ng g^{-1})	Brown rice (n = 90) (ng g^{-1})
Mean	100.17	80.12
95% CI	89.02 – 111.31	68.49 – 91.76
Median	94.85	72.54
Standard deviation	44.62	55.56
Variation	1991.24	3086.95
Minimum	4.30	4.26
Maximum	228.70	240.89

Table S3. Estimated average daily dose (mg/kg-day) associated with exposure to inorganic arsenic in polished (white) and husked (brown) rice.

Age (years)	White rice		Brown rice	
	Mean	95th percentile	Mean	95th percentile
< 1	1.01×10^{-04}	1.73×10^{-04}	4.01×10^{-06}	5.63×10^{-06}
1	4.27×10^{-04}	7.35×10^{-04}	1.70×10^{-05}	2.39×10^{-05}
2	3.44×10^{-04}	5.91×10^{-04}	1.37×10^{-05}	1.92×10^{-05}
3	3.36×10^{-04}	5.78×10^{-04}	1.34×10^{-05}	1.88×10^{-05}
4	3.27×10^{-04}	5.61×10^{-04}	1.30×10^{-05}	1.83×10^{-05}
5	2.57×10^{-04}	4.41×10^{-04}	1.02×10^{-05}	1.44×10^{-05}
6	2.37×10^{-04}	4.07×10^{-04}	9.43×10^{-06}	1.32×10^{-05}
7	2.10×10^{-04}	3.61×10^{-04}	8.37×10^{-06}	1.18×10^{-05}
8	1.89×10^{-04}	3.26×10^{-04}	7.55×10^{-06}	1.06×10^{-05}
9	1.66×10^{-04}	2.85×10^{-04}	6.61×10^{-06}	9.27×10^{-06}
10	1.45×10^{-04}	2.49×10^{-04}	5.78×10^{-06}	8.11×10^{-06}
11	1.29×10^{-04}	2.21×10^{-04}	5.13×10^{-06}	7.20×10^{-06}
12	1.14×10^{-04}	1.96×10^{-04}	4.54×10^{-06}	6.37×10^{-06}
13	1.03×10^{-04}	1.77×10^{-04}	4.10×10^{-06}	5.76×10^{-06}
14	9.57×10^{-05}	1.65×10^{-04}	3.82×10^{-06}	5.36×10^{-06}
15	8.93×10^{-05}	1.54×10^{-04}	3.56×10^{-06}	5.00×10^{-06}
16	8.63×10^{-05}	1.48×10^{-04}	3.44×10^{-06}	4.83×10^{-06}
17	8.34×10^{-05}	1.43×10^{-04}	3.32×10^{-06}	4.67×10^{-06}
18	8.12×10^{-05}	1.40×10^{-04}	3.24×10^{-06}	4.54×10^{-06}
19	8.31×10^{-05}	1.43×10^{-04}	3.31×10^{-06}	4.65×10^{-06}
20 to <25	7.98×10^{-05}	1.37×10^{-04}	3.18×10^{-06}	4.46×10^{-06}
25 to <30	7.62×10^{-05}	1.31×10^{-04}	3.04×10^{-06}	4.27×10^{-06}
30 to <35	7.48×10^{-05}	1.29×10^{-04}	2.98×10^{-06}	4.19×10^{-06}
35 to <45	7.38×10^{-05}	1.27×10^{-04}	2.94×10^{-06}	4.13×10^{-06}
45 to <55	7.31×10^{-05}	1.26×10^{-04}	2.92×10^{-06}	4.09×10^{-06}
55 to <65	6.79×10^{-05}	1.17×10^{-04}	2.71×10^{-06}	3.80×10^{-06}
65 to <70	3.22×10^{-05}	5.53×10^{-05}	1.28×10^{-06}	1.80×10^{-06}
